# Family listening/circle program: The experience of community action projects to promote family and community wellness in three tribal communities in New Mexico

**DOI:** 10.3389/fpubh.2023.1091751

**Published:** 2023-03-30

**Authors:** Rebecca Rae, Lorenda Belone, Eleanor Tafoya, Melissa Yepa, Benalda Cohoe-Belone, Ira Burbank, Ardena Orosco, Pius Lacroix-Garcia, Mingma Sherpa, Nina Wallerstein

**Affiliations:** ^1^College of Population Health, Center for Participatory Research, University of New Mexico, Albuquerque, NM, United States; ^2^College of Education, University of New Mexico, Albuquerque, NM, United States; ^3^Department of Education, Jemez Language Program, Jemez Pueblo, NM, United States; ^4^Department of Education, Hemish Pilot Immersion School, Jemez Pueblo, NM, United States; ^5^Ramah Navajo School Board, Inc., Pine Hill, NM, United States; ^6^Behavioral Health Services, Ramah Navajo School Board, Inc., Pine Hill, NM, United States; ^7^Mescalero Prevention Program, Mescalero, NM, United States; ^8^School of Medicine, University of New Mexico, Albuquerque, NM, United States; ^9^College of Population Health, University of New Mexico, Albuquerque, NM, United States

**Keywords:** community-based participatory research, Indigenous community-engagement, culture centered intergenerational prevention program, community action project, empowerment, American Indian

## Abstract

**Introduction:**

The Family Listening/Circle Program (FLCP) is a community-based participatory research (CBPR), culture-centered, intergenerational family strengthening program that was co-developed in partnership with the University of New Mexico's Center for Participatory Research (UNM-CPR) and three tribal communities (Pueblo of Jemez, Ramah Navajo, and Mescalero Apache) in New Mexico. The Family Listening/Circle Program brings together fourth and fifth graders, their parents, caregivers, and elders to reduce risky behaviors associated with the initiation of substance use among the youth, and to strengthen family communication and connectedness to culture and language as protective factors.

**Methods:**

The tribal research teams (TRTs) from each community worked with UNM-CPR to co-create, pilot, implement, and evaluate the tribally-specific FL/CP curricula centered in their own tribal histories, language, knowledge, visions, and actions for the future. A key component of the FL/CP involved the planning and completion of community action projects (CAPs) by participating families. During the final session of the program, the families present their community action projects on poster boards, with children leading the presentations. The TRTs and UNM team document narratives of what was shared and learned by the families.

**Results:**

The CAPs provide an empowerment and community benefit focus based on Paulo Freire's philosophy that people can become agents of change if they identify and work on issues that are important to them. The community action projects are also centered in Indigenous values and practices of reciprocity, responsibility, and being active members of the community.

**Discussion:**

The CAPs added unique contributions to the Family Listening/Circle Program as the participants' learnings were strengthened when they had the opportunity to give back to their communities. The CAPs were important to document as they illustrated the potential range of effectiveness with their capacity to empower participants to address challenges within their communities, strengthen cultural norms and values, and improve the wellbeing of community members.

## Introduction

Research with American Indian/Alaska Native (AI/AN) communities has increasingly turned to community-based participatory research (CBPR) approaches to reduce inequities and strengthen wellness among AI/AN populations. Tribal oversight and participation in research have grown in the past two decades to counter historic abuses of “helicopter” research, negative stereotyping, and disregard for community knowledge and participation. Tribes now expect and demand the benefits of studies, including ownership of data and authority over publication. In the last decade, guidelines specific to AI/AN communities have been more defined, with recognition of tribal sovereignty and use of the term *Tribal Participatory Research* (1–4); or *Indigenized Research* (5–8).

In New Mexico (NM), three American Indian tribes came together to engage in a National Institutes of Drug Abuse/National Institutes of Health (NIDA/NIH) funded R01 5-year CBPR research study with the University of New Mexico Center for Participatory Research (UNM-CPR) to evaluate the effectiveness of the Family Listening/Circle Program (FL/CP), a culture-centered and evidence-based intergenerational family prevention program. This collaborative research has been a long-term commitment and partnership which has sought to honor tribal direction and ownership of research through multiple NIH funding cycles (9–12).

The Family Listening/Circle Program brings together fourth and fifth graders, their parents, and elders to reduce risky behaviors associated with the initiation of substance use among the youth, and to strengthen family communication and connectedness to culture and language as protective factors. A key component of FL/CP involves the planning and completion of community action projects (CAPs) by families, which provide an overarching empowerment and community benefit focus. The premise of community action projects is centered on Indigenous values and practices of reciprocity, responsibility, and being active members of the community. Drawing from Paulo Freire's liberatory listening/dialogue/action educational methodology (13), community action project ideas emerge from listening to youths' concerns about their community, engaging them in dialogue with their families for support, and creating collective actions that empower them toward community improvement (14–17).

While AI/AN communities are particularly at risk for health disparities, facing high rates of intergenerational trauma, as well as structural inequities, such as high unemployment, they also have significant cultural and language strengths to inform research. Much evidence demonstrates that connection to history, land, language, traditional food, and cultural practices have a positive impact on Indigenous health (18–20). With Indigenous culture inextricably linked to land and place, a collectivist sense of community and self emerges that can promote healing from discrimination and negative trauma from assimilative policies (7, 21, 22).

Connection to culture and community can also facilitate civic participation (23, 24). There is evidence that these connections may decrease stress, increase adaptive psychosocial resources and reduce the likelihood of negative outcomes (such as anti-social behaviors) in the long term (16, 25). In Australia, the most commonly used definition of health for Indigenous peoples states that health is “not just the physical wellbeing of an individual but is the social, emotional and cultural wellbeing of the whole community” (17), nurturing relational restoration *via* worldviews across body, place, self, family, community, past and future generations (7).

An additional concept of culture-centeredness proposes that for interventions to be effective and sustainable, they must recognize culture not just as a set of beliefs, but as people's agency, voice, and power to direct the changes needed in their community (2, 26). Shared principles include the right of Native peoples to base research in their own knowledge and priorities, and to participate in research processes based on dialogue, longer timeframes, decolonized methodologies, culture-centered interventions, and recognition of tribal diversity (18, 27–29). Multiple governance mechanisms, such as tribal or intertribal IRBs, tribal research committees, tribal councils, and other leadership oversight, have begun to codify these benefits and principles (30–32).

The Family Listening/Circle Program has embraced this comprehensive understanding of culture by advancing the research practice of calling community advisory committees to be called *Tribal Research Teams* (TRTs), to honor tribal community partners' equal status as co-researchers (9, 33). The TRTs have worked together with UNM-CPR for over 14 years to co-create, pilot, and now implement three tribal-specific Family Listening/Circle Program curricula, based on their own tribal histories, language, knowledge, visions, and actions for their future. The authors for this article included six tribal partners (Jemez Pueblo, Ramah Navajo, Mescalero Apache), two from each of the three tribal communities (two teachers and four service providers) and four from the academy, two co-principal investigators (Navajo and Jewish), one Native co-investigator researcher (Jicarilla Apache) who is leading the writing team and an international graduate student (Nepalese). We started this article at a bi-annual meeting with the tribal partners and UNM team brainstorming ideas, which became an iterative and active process, sharing and discussing working drafts at numerous meetings.

For this article, we first present the background of each tribal community and their long-term research partnership with UNM-CPR, which has ranged between 14 to 20 years. Secondly, we summarize the CBPR process to co-develop three evidence-based and tribal/culture-centered family curricula with blended Indigenous theory and Western behavior change theory, rather than being simply a “tailored” program. Thirdly, we showcase the community action projects that demonstrate how children and parents deepened their involvement within their community and culture to become advocates and change agents. Finally, we end with results, limitations, and recommendations for other intervention programs, interested in incorporating cultural-centeredness, empowerment, and community action projects to promote health equity and community health.

## Community-based participatory research

**Figure 1:**
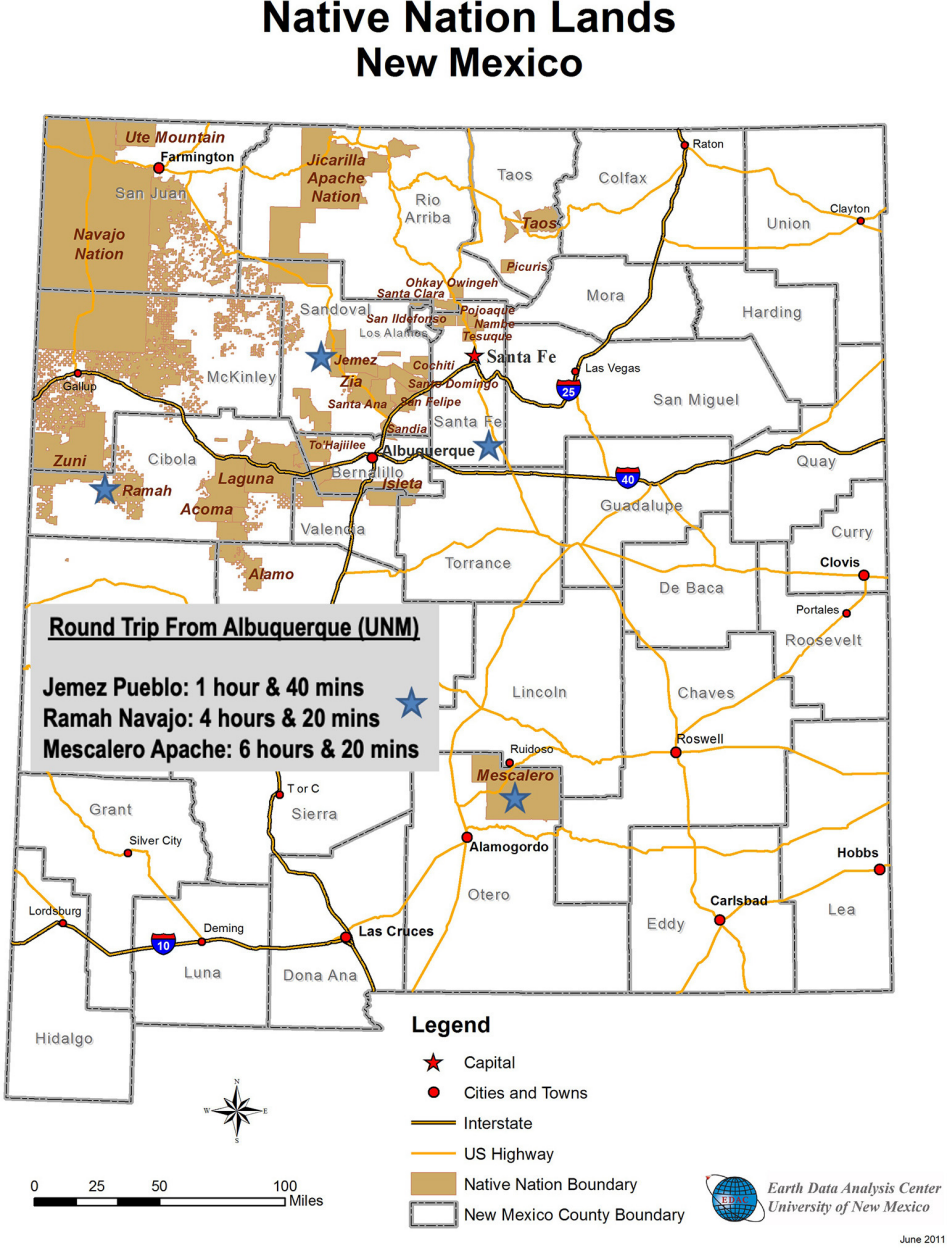
Map of New Mexico tribes and location of three tribal community partners.

The UNM Center for Participatory Research was founded on CBPR principles, defined “not simply as a community outreach strategy but rather a systematic effort to incorporate community participation and decision making, local theories of etiology and change, and community practices into the research effort” [http://cpr.unm.edu/index.html, (32, 34, 35)]. The tribal community partnerships were initiated individually between UNM-CPR and each tribe. This grew into a collective collaboration, focused on the co-implementation and testing of a culture-centered intervention through the shared NIDA grant (2009–2014), facilitating three tribal-specific Family Listening/Circle Programs with common curricular elements, even as each tribe retained its own cultural knowledge, history, and values. The Family Listening/Circle program works with elementary children to strengthen resiliency and increase protective factors, such as cultural identity, language, anger management, and communication, to hinder alcohol and substance abuse initiation. According to the literature (36–38), youth that delays the first initiation of alcohol and substances are more likely to not experiment or develop addictions to alcohol or substances. The three communities, all committed to improving the lives of Native children and families, represent New Mexico's three dominant tribes of Pueblo, Navajo, and Apache. Figure 1 provides a map with the locations and representation of tribes in New Mexico.

### Pueblo of Jemez

The Pueblo of Jemez is a federally-recognized tribe traditionally known as *Hemish of Walatowa* located 1-h northwest of Albuquerque, with community members living in a single village known as Walatowa and encompassing over 89,000 acres of land. Of the ~3,400 tribal members, 58% live in Walatowa (http://www.jemezpueblo.org) (39) and are the only Towa-speaking community, with language fluency, last surveyed in 2006, was 80% across all generations (40). To address language, the Tribal Council passed a resolution in December 2012 to convert their Head Start Program to full language immersion and in 2016 the Walatowa Head Start Language Immersion Program was completed. Jemez was the first tribe to charter two schools under the NM Charter School Law: San Diego Riverside Charter School was established in 1999 and the first on tribal lands, Walatowa High Charter School, the first Native high school chartered in 2002 and the second on tribal lands. Both schools are unique in that they were approved by Tribal Council resolution to exist as state-funded public charter schools on tribal trust lands with language and culture-based curriculum and programs at the core of both charters, locally determined with strong community and tribal input. In 1999, Jemez also took over its health care system from the Indian Health Service and broadened its focus to include prevention programs focused on healthy traditional foods (i.e., growers' market, nutrition classes) and physical exercise (i.e., running and cycling clubs) (10).

The Pueblo of Jemez and UNM-CPR partnership was initiated in 1999 and has since collaborated on four CBPR research studies. The first in 1999 from the Centers for Disease Control and Prevention (CDC), the “Community Voices” research study partnership ran from 1999 to 2003 and identified cultural strengths and community capacities as a means of understanding social capital in an indigenous context (41). Based on the “Community Voices” recommendations, the partnership successfully obtained a Native American Research Center for Health III (NARCH) pilot grant (2005–2009) to co-develop a culture-centered family intervention to reduce child risk factors using Hemish cultural strengths (10, 12). Building from an existing Anishinabe curriculum (http://www.ppsi.iastate.edu/american-indian-prevention) (42), the Jemez community advisory committee expanded its membership to include elders, who provided history and language knowledge for re-centering the curriculum in Hemish culture. After 2 years of development, the Hemish of Walatowa Family Circle Program (FCP) curriculum was finalized and piloted with two family cohorts. Poised for larger testing of FCP, Jemez joined the successful R01 NIDA application for their third and largest collaborative research study. With other collaborative initiatives, the community advisory committee redefined itself as a Tribal Research Team (TRT) to own co-researcher status (9). Transitioning to TRT signified that tribal community partners were no longer in an “advisory” role; they were active and engaged researchers directing the project to meet their community needs. See TRT timelines for the evolution of their partnership at http://cpr.unm.edu/research-projects/flcp/historical-timelines.html.

### Ramah Band of Navajo

The Ramah Band of Navajo is located in Western New Mexico 2.5 h west of Albuquerque. Ramah Navajo encompasses over 170, 000 acres and is one of three non-contiguous satellite reservations of the main Navajo Nation Reservation and is located in a “checker-board” area based on the fact that the land status in and around Ramah Navajo includes tribal trust lands, individual American-Indian allotments, privately-owned, and state lands (i.e., Bureau of Land Management). Hundreds of Ramah Navajo community members were forcibly removed in the 1860's “Long Walk” by the U.S. Government and lands had to be reclaimed throughout the 1940's (43). Not until 1955 was Ramah Navajo officially recognized as a Chapter, one of 110 local governing bodies of the Navajo Nation (www.ramahnavajo.org) (44, 45). Ramah Navajo was the first tribal community in New Mexico to exercise self-determination and established its own educational system in the 1970's, under Public Law (PL) 93–638. At the same time, the Ramah Navajo School Board (RNSB) was established as a non-profit to oversee the new tribal school and in 1978 RNSB expanded its role to run its own clinic and health and social services department from the Indian Health Service (11, 44). Today, Ramah Navajo's enrollment is estimated at over 3,500, with over 400 students from Head Start through 12th grade enrolled in the local school.

The Ramah Navajo and UNM-CPR partnership began in 2000 and has since collaborated on four research studies. The first study was a CDC grant received by the Albuquerque Area Indian Health Board (AAIHB), to increase cervical and breast cancer screening in the community of Ramah Navajo. Based on that experience a second study was obtained, a NARCH I (2001–2005) focused on the creation of a comprehensive community profile to assess community capacity as well as historic losses and a range of health, education, and community issues (46, 47). This study was guided by what was called the Ramah Navajo Advisory Board to guide the community capacity dimensions, focusing on youth, elders, sense of community, culture, communication, women, and leadership (31). Data analysis from 250 households led to findings, similar to Jemez's Community Voice's findings, that cultural preservation was highly valued, but concerns remained about language loss and family communication breakdown.

The next research grant for Ramah Navajo was the NARCH III pilot grant (2005–2009), collaboratively with Jemez, to co-create their family curriculum (i.e., Family Listening Program) grounded in their culture and language context. Piloting of their FLP was overseen by the Ramah Navajo Advisory Committee (RNAC), similar to the council established in their first study. The RNAC conducted one pilot with 10 families (10), and based on the very positive experience, Ramah Navajo joined the R01 NIDA application for their third research study. Similar to Jemez, the RNAC redefined itself to what was called as a Tribal Research Team (TRT) to own their co-researcher status, with their partnership timeline showing their evolution (http://cpr.unm.edu/research-projects/flcp/historical-timelines.html).

### Mescalero Apache tribe

Mescalero Apache is a federally-recognized tribe with more than 5,000 enrolled members located in south-central Sacramento Mountains, 3 h south of Albuquerque. The reservation was established in 1873 and covers 440,000 acres. There are three tribal sub-bands, Mescalero, Chiricahua, and Lipan Apache, and the language spoken is Southern Athabaskan. Four sacred mountains are within Mescalero's homelands: The Sierra Blanca, Guadalupe Mountains, Three Sisters Mountain, and Oscura Mountain Peak. A leader in Native American sovereignty issues in water rights and business, Mescalero Apache hosts several tribal enterprises, i.e., Ski Apache, Inn of the Mountain Gods Resort and Casino, Mescalero Forest Products, Mescalero Gas Company, and Mescalero Apache Telecom (www.mescaleroapachetribe.com) (48). The Mescalero Apache School, kindergarten to 12th grade (K-12), is operated by the Bureau of Indian Education with about 550 students enrolled. Before 1995, Mescalero Apache had only a day school for elementary students and middle and high-school students were required to commute to neighboring non-Native communities. Under the late Tribal President Wendell Chino, the current K-12 school was built with the first graduating class in 1996. Health services are still provided under the Indian Health Services. In 2009, Mescalero Apache entered into a research partnership with UNM-CPR and has since collaborated on two NIH-funded research studies. The FLP was the first tribally-directed culture-centered prevention program to target youth before substance abuse experimentation (49).

Mescalero Apache learned of the Family Listening Program through a NARCH III presentation to the Albuquerque Area Indian Health Board (AAIHB) which was the administrative center for the NARCH projects in New Mexico. In that meeting, one of the AAIHB advisory members was from Mescalero and she regularly heard the reports about the Family Listening/Circle Program with Jemez Pueblo and Ramah Navajo. The advisory member then requested that FLP be brought to her community and with approval from her Tribal Council, UNM-CPR and Mescalero Apache co-submitted, and received NARCH V pilot funding from 2009 to 2014 to create their family curriculum (i.e., Family Listening Program) grounded in Mescalero Apache history, values, and knowledge. Mescalero Apache established its own community advisory committee, yet midway through the grant changed to a TRT (49). The TRT was able to pilot their family program twice and based on their experience joined the R01 NIDA application for their second research study. Similar to Jemez and Ramah Navajo, the Mescalero Apache partnership timeline can be viewed at http://cpr.unm.edu/research-projects/flcp/historical-timelines.html.

## Methods

Through a CBPR process, the FL/CP curriculum was co-developed with each tribe incorporating their own cultural teachings, histories, and community learnings into their unique tribally-centered curriculum, while retaining similar indigenized cognitive-behavioral evidence-based strengthening family components across all curricula. While supporting external validity through shared components, each tribal research team was able to integrate their own teachings and make the Family Listening/Circle Program their own. Each tribal community contracted a local community artist to design distinctly different program logos, illustrations, and images meaningful to their community and significant cultural teachings. The curriculum also includes an empowerment focus based on the philosophy of Paulo Freire (13), that individuals/families can become agents of change if they identify and work on issues that are important to them (50). This empowerment perspective incorporated sessions for children, parents, and elders to create their own visions, identify community challenges, and choose a community action project to address community challenges. Children were given cameras or used their camera phones to create a photovoice documentation of their CAPs, sharing their photos to promote further community dialogue (51, 52).

While each curriculum is unique, they follow the same structural format that begins with prayer, followed by dinner, traditional introductions (clan, Indian name, or welcome) with practice in their tribal language, a review of home practice, main content experiential exercises (often in separate children and adult groups), group dialogue, journaling, and wrap-up with a small incentive. The first six sessions of the curriculum are grounded in teachings specific to each tribe and incorporate cultural introductions, cultural foods, families eating together, relationships (clanship, kinship), core values, cultural family roles, ancestral lineages of the people, phases of life, cultural responsibilities and ceremonies linked to those phases, and visions for the community. Sessions 7–12 include indigenized cognitive-behavioral exercises that help strengthen communication, help-seeking, anger management, problem-solving, exploring discrimination while highlighting the beauty in differences, positive relationships, empowerment, and building social support within community networks. Table 1 provides an outline of the curriculum sessions, objectives, and introduction example.

**Table 1:** Family listening/circle program curriculum.

**FL/CP curriculum sessions, objectives and introduction example**	
**Session 1: welcoming** • Welcome participants to Family Listening/Circle Program and reflect on values • To review the guidelines and goals of the program over the next 12 weeks “*The FLP/CP is to support healthy families and learn our culture and language. Some activities we'll do include sharing a meal, discussing topics with your family, and developing a community action project*	**Session 7: community challenges** • To identify areas of concern in the community • To identify people and groups who can support our efforts to improve the community through community action projects “*Session seven will help us identify challenges we face as a community and to begin exploring solutions. We will discuss what challenges we could address through a community action project”*
**Session 2: tribal history (Part I)** • To increase knowledge and pride in tribal community history and strengths as a people • To help build and strengthen tribal identity “*We will learn our tribal history and how our customs and traditional ways are passed on through families. Knowing our history and where our people came from can create a sense of pride in who we are. Our people have lived through much struggle and joy”*	**Session 8: communication, help seeking, and problem solving** • To learn how to communicate better, to seek help, and problem solve • To learn how to talk about emotions and reinforce emotional reactions using tribal community values “*Session eight will address communication, asking for help, and problem-solving using role playing and skill building scenarios”*
**Session 3: tribal history (Part II)** • To increase knowledge and pride in tribal community history and strengths as a people • To help build and strengthen tribal identity “*We will learn our tribal history and how our customs and traditional ways are passed on through families. Knowing our history and where our people came from can create a sense of pride in who we are. Our people have lived through much struggle and joy”*	**Session 9: recognizing types of anger and managing anger** • To understand anger as a normal emotion • To identify factors that contribute to anger • Learn ways to manage anger using cultural values “*We will discuss what anger is and where it comes from and learn how anger can be harmful to our health. We will discuss how to manage anger in a healthy way”*
**Session 4: my family** • To reflect on family, community, and cultural strengths and practice active listening • To learn about respectful and positive communication “*We will explore ways to communicate to bring family together to learn, discuss, and make decisions in a respectful way. W*e *will practice listening to one another* and *will learn about our family trees”*	**Session 10: being different and positive relationships** • To understand that differences make us unique, not unequal as human beings • To challenge stereotypes and appreciate diversity • To understand the value of recognizing our own biases and how they affect our actions “*We will talk about being different and finding our strengths in differences. We will discuss how to have respectful conversations among diverse groups”*
**Session 5: tribal way of life** • To recognize the importance of role models at home and in the community • To learn important cultural roles and responsibilities with each phase of life “*Our way of life can reflect what you believe in, values your family practice, language spoken, or the daily habits/activities practiced in our community”*	**Session 11: building social support** • To identify supportive people in participants' lives • To learn how to build a support system • To build pro-social and pro-active peer support “*In this session we will focus on building and strengthening our social support. Social support means identifying people we trust and who we can count o.”*
**Session 6: our vision** • To engage in community visioning • To create a personal vision for the future • To begin discussing community action projects “*In this session we will practice creating a personal and community vision for our future. We will discuss ways we can give back to our community through a community action project”*	**Session 12: making a commitment and community project presentations** • To conclude the 12-week program and share community projects and lessons learned • To evaluate the program “*This session will conclude our 12-week program. Everyone will share their community action projects and highlights of the program”*

The Family Listening/Circle Program is implemented in each tribal community by trained community TRT facilitators. Facilitators have included teachers, behavioral health staff, prevention specialists, public health educators, parents, and elders. The facilitators were instrumental in helping families plan, organize, and implement their CAPs. The TRT facilitators let families know the goal of the CAP is to contribute to community improvement without the expectation of solving huge community problems. Families were encouraged to envision projects that were feasible within the timeframe of the program. Program participants had the flexibility to complete their project either as an individual family or as a group of families. The community action projects are introduced in session five and six when program participants discuss their community visions and the challenges they see in their community. As children and families identify community challenges, the facilitators help them think through potential solutions to address their concerns.

Ideas about addressing challenges from the children included: picking up trash; gathering items for ceremonies; doing food and clothing drives; bridging gaps between themselves and elders; substance abuse awareness; and ways in which they could give back to their communities. Based on these discussions, the families were asked to select an area of improvement in the community they would like to address and they develop a plan of action to complete their community action project by the end of the program. The FL/CP program provides funding to support the families' community action project efforts. As part of the R01 CBPR research study, each Tribal community received its own budget to support running the FL/CP program (staff time, food, supplies, incentives, etc.), paying facilitators, and implementing the community action projects. The Tribal community PIs and TRTs determine the budget for the CAPs and they help the families stay within budget. However, the majority of the CAPs had minimal costs, mainly for supplies such as trash bags, paint, and tools.

The families plan out their CAPs through the last six sessions with guidance and support from the TRT facilitators. When the CAPs were implemented varied among the communities and families, particularly if the CAPs were completed by individual families or as a group. However, generally, the CAPs were completed within several weeks after the eleventh session of the curriculum. The twelfth session is held after the families complete their CAPs, as the last session provides time for families to present their community action projects on poster boards, with children leading the presentations. The TRTs and UNM team document what was shared and learned by the families.

## Results

### Pueblo of Jemez

After four waves of implementation of the Family Circle Program, four cycles of CAPs were completed, with families tending to engage in individual family CAPs. Examples include: one family deciding to clean up the path from their home to their child's school. Even though the cleanup day was very windy, the children still wanted to complete the project, highlighting how upset children were about the trash being thrown onto community roads and their willingness to be out in harsh conditions for the cleanup. Another family decided to update the community bulletin board, a place where community events were posted. The board had many old flyers, but after the cleanup, the board received an updated look with current information. Another family decided to collaborate with the Jemez Department of Planning and Transportation to post a speed limit sign on a community road, which had increased traffic where children played. After posting the sign, a community member commented, “Yes, I have seen several [speed limit signs] in several places that were not there before,” implying a much-needed change in the community.

Other examples of CAPs included the cleanup of the village plaza after a Feast Day event, the cleanup of neighborhoods, and the posting of speed limit signs near school zones. The TRT and community members have seen families involved in CAPs become empowered through their participation. One community member stated, “It [CAPS] is to improve community life, so I think that's a good way to start teaching them [children] about stuff like that, it's really important.” In each year of the last session of the program, Jemez community and tribal council members have been invited to the presentations and families have showcased their CAPs, which children conducted in their Towa language.

### Ramah Navajo

Program families in Ramah Navajo have tended to come together as a group to organize and implement their CAPs. For example, during one wave of program implementation, the participants decided to conduct a group cleanup of the community fitness trail, which had been neglected for years. Families initiated the planning, spoke on the local radio, and created flyers to invite community members to join in the cleanup effort. In the planning, a unique collaboration was formed between the Tribal Security, Health Promotion and Education, and the radio station to accomplish the project. 2 years later, the fitness trail is still being maintained and used by community members.

During another year, some participants conducted individual CAPs, driven by individual child interests; and others teamed up for a group Elder Food Drive. Often students have big ideas for their projects, which require more funding for completion. However, the facilitators assist in the narrowing of the projects to assure realistic and achievable goals. In the last year of the FLP funding, the CAPs goal to empower students was reached when students presented their project at the Ramah Navajo Chapter House. One of the high school student interns who was hired to assist in the Family Listening Program was encouraged to run for the Ramah Navajo Queen Contest, a platform that allows students to address community concerns. The intern won the competition and was able to give voice to the FLP children by sharing community needs, enabling her to work toward strengthening community bonds, while improving her communication skills and instilling Ramah Navajo pride and identity.

During another year families came together and hosted an event to collect donations (clothing, household items) to distribute to families in need in their community. With substantial donations, after families came and took items they needed, leftover items were donated to the Social Services Program. Another group of families focused their CAP on promoting positive messages for the community. The families created signs with positive messages and placed them at intersections for people to see. The families created the signs to get people thinking about drug and alcohol use. The resiliency-based messages at the different intersections stated, “Doing good does you good,” “Love your family, NOT alcohol!” and another quote that addressed methamphetamine use.

### Mescalero Apache

In Mescalero, four CAPs were completed, with grant funding providing support for supplies and equipment. During the first round of planning, the first group of families opted to complete a group CAP to lessen the burden on individual families. Through a consensus process, the families decided to clean up several local lake recreation areas. They created hand-painted signs that asked community members to keep the lake clean and not litter, with signs posted in picnic areas. Photos were taken with the children proud of their artistic contribution to the community. The TRT stated that they heard community members say they were happy to see the signs posted around the lake. To date, only one sign was vandalized but the other signs remain in good condition. A TRT member stated, “This shows that the community appreciates and values the meaning of the signs.” For the second family cohort which took place under winter snowy mountain conditions, the families sought a community action project inside. From the brainstorming of ideas during the FLP session, the families decided to contact local clergies and offered to clean up their church. A couple of the church pastors were surprised, and slightly puzzled by the offer because it was something they were not used to. Two churches accepted the offer and the families split into two groups. One group focused on cleaning the inside of the church, while the other group focused on cleaning up outside the church, bundled up against the weather. While people in the community were surprised when the FLP participants offered to clean up the churches, the families felt there were many different avenues to giving back to your community and they wanted to pitch in.

Other CAPs that the families organized and implemented were a canned food drive and a Christmas meal for the community. For the food drive, families reached out to the community for food donations. Once the families collected enough donations, they organized the food into bags. Altogether the families distributed 75 bags of food to residents of Mescalero. Another group of families decided to host their CAP during the holiday. Typically, the CAP is done in the springtime, but the program families wanted to host a community meal for Christmas. The families came together and cooked the Christmas meal and served over 350 meals to the community.

## Discussion

The CAPs have added unique contributions to the Family Listening/Circle Program. The children and parents learned together about their history, community values, and ways of life during the dinner-based program, and their learnings were strengthened when they had the opportunity to give back to their communities. The CAPs were important to document as they illustrate the potential range of effectiveness with their capacity to empower participants to address challenges within their communities, strengthen cultural norms and values, and improve the wellbeing of community members. Evidence of CAP effectiveness is documented through the taking of photos by the child participants during their CAPs activity. During the final session of the program, the children along with their families present what they learned in the program and they share their CAP experiences. The children create poster boards and display photos they took throughout the program and of their community action project. The photos helped the children talk about their experiences as they could describe what was happening in the photos. The photos helped trigger their memories and generated excitement as they expressed pride in what they accomplished.

The CAPs contributed to outcomes at multiple levels: individual self-confidence, skills in group decision-making and consensus-building, a sense of community empowerment, cultural pride, as well as the transformation of community environments and perceptions of the leadership. Participating in the program and learning about their cultural traditions and language empowered the children to be active participants in choosing their CAPs. The CAPs provided opportunities for the children to see their community with new eyes, to become more aware of their surroundings, and to voice solutions to the problems they were experiencing. The parents in the program were impressed by what their children noticed and voiced about the challenges in their community as well as their ideas to improve those conditions. The range of CAPs outcomes is documented here:

### Personal

New skills and self-confidence have been displayed. In Ramah Navajo, the FLP high school student helpers conducted radio PSAs for the first time, one ran for the title of Ms. Ramah Navajo, and all gained skills in leading discussions with younger children. While initially less willing to use their Native language during sessions, most children in Jemez Pueblo gave their final CAP presentation in the Towa language. Participating adults have said that they have seen a change in themselves: we are “more aware of community concerns, issues with political and cultural activities. Community members want to change these.”

### Social support and team building

The CAPs facilitated opportunities for team building among the families. Families that decided to do group projects had to create consensus on what they were doing, identify materials/supplies needed, plan out dates/times, and show up to do the work. The community clean-ups, community food drives, and community meals strengthened the families' support of one another and their community. During the session on community concerns, both adults and children identified community issues, and ways to seek support to address them. One participating adult wrote in the journal, “There are many similarities in the concerns of community members which is eye opening, yet you feel stronger to hit these concerns head on knowing that you have the support of those around you.” Another wrote, “If we all work together, we can improve our community. We can create change.”

### Culture and history pride

In all communities, families felt their community projects re-emphasized to them the importance of their land and culture. In Mescalero Apache, families wanted to clean up their lakes to represent cultural pride in their homelands. In Ramah Navajo, for example, children realized the elder who spoke to them about history was part of the photograph on the wall of tribal leaders who took back control of their school in the 1970's from the federal government. Participating students chose to wear traditional dress for their final presentation of their CAP. Giving back to the community has deepened cultural identity and pride in Jemez, with more children dancing at feast days, and strengthening connections to their families, a key protective factor as they grow older.

### Community program collaboration

The community action projects helped facilitate new collaborations between community programs and groups. In Jemez, one of the families collaborated with the Jemez Department of Planning and Transportation to post a speed limit sign. The family had to meet with the department of planning and transportation to discuss their CAP proposal and the need for a speed limit sign in a designated area where a lot of children play. The Ramah Navajo fitness trail cleanup involved collaboration with the radio station, school, and security department. The families worked with the radio station to advertise the community trail clean-up and partnered with the school for supplies. In Mescalero, the families collaborated with the church community.

### Community leaders

In Jemez, multiple leaders (fiscales, administration, governors) have joined the last session of the program and spoken about how proud they were of the children, stating what they learned in the Towa language. A couple of Tribal Council members attended the community Christmas meal that the Mescalero Apache FLP families hosted. They acknowledged the program for hosting the community meal. A former Mescalero Tribal Council member became a TRT member and as an elder fluent in the language was instrumental in teaching the language in the program. The Ramah Navajo School Board welcomed FLP families to present to them, listened to their concerns, and has been in support of the FLP program.

### Community benefit and dissemination

The CAPs have had some lasting impacts in the respective communities. For example, two out of the three Mescalero Apache signs at the different lakes are still up providing messages to keep the land clean. In Ramah Navajo, families continue to upkeep and use the fitness trail years later. In Jemez, children express pride that their speed limit signs are up, that they've cleaned up areas, and that they can create bigger visions.

The purpose of the CAPs was to demonstrate to children (and adults) that they can be change agents and be empowered in their own communities to achieve successes, even if small, and build future leadership skills and confidence. In all communities, the CAPs were recognized with photos, articles in the local newsletters, and community recruitment flyers that included information on the CAPs. All three communities shared the challenge of devising CAPs that were feasible and doable within the time frame of the program. Some families and children had big visions, which resulted in families being overwhelmed or intimidated by the project. However, the TRT facilitators assisted families to narrow CAPs to smaller projects and provided support as needed.

The TRTs shared their experiences with implementing the program and the CAPs during bi-annual in-person meetings that were held as part of the research project. At these meetings, the TRTs discussed what processes worked for their community, adaptations they made to make the CAPs feasible, and how they problem-solved the challenges they faced. Through this cross-learning, the TRTs gained new ideas to strengthen how the CAPs came together in their community. TRT members from the three communities also co-presented at several conferences where they continued to learn from one another. For example; through the learnings of implementing the program and CAPs, the TRT facilitators started to introduce the CAPs earlier in the program to address the time constraints. In the Pueblo of Jemez, for example, facilitators have chosen to show pictures of previous CAPs during the first introductory session to prepare new program families to think about possible projects. Mescalero Apache focused on group projects because single-family projects can be daunting and the other communities started to provide a group option for their families.

The Family Listening/Circle Program strengthens identity through the cultural teachings and language which are protective factors and facilitators for strengthening coping, resiliency, and hope (15, 17). The literature also supports that a strong cultural identity strengthens a connection to community, a sense of place, and civic engagement, which can decrease stress and increase adaptive psychosocial (16, 25). The community action projects coupled with the teachings from each program session (communication, help-seeking, anger management, etc.) had a positive impact on the youth as indicated by preliminary effectiveness data. Promoting communication and encouraging group work, the CAPs have highlighted the benefits of families giving back to the community. Children and adults that collaborated during the planning and implementation of the CAPs have learned new skills, built self-confidence, found support among other FL/CP participants, strengthened their connection to their culture, and gained a new sense of empowerment in being able to see their accomplishments at a community level. The CAPs have expanded the program goals beyond individual family strengthening to providing service and benefit to their communities.

In sum, community action project outcomes mirror the importance of cultural connections and community benefits within the participatory community engagement literature on Indigenous youth (24). Families working together through dialogue and action deepened community capacities to promote cultural identity, connection to tribal lands, and health (14), which was a major aim of the Family Listening/Circle Program. While this article did not share program outcome data, the program has preliminary effectiveness data that shows a decrease in anxiety and depression among child participants (9). The UNM-CPR and TRTs are co-writing another manuscript to share the rigorous intervention effectiveness data.

## Data availability statement

The raw data supporting the conclusions of this article will be made available by the authors, without undue reservation.

## Ethics statement

The studies involving human participants were reviewed and approved by University of New Mexico IRB, Navajo Nation IRB, Southwest Tribal IRB. Written informed consent to participate in this study was provided by the participants' legal guardian/next of kin.

## Author contributions

The UNM team RR, LB, MS, and NW contributed to the introduction, research, literature, results, and discussion. The tribal community partners ET, MY, BC-B, IB, PL-G, and AO contributed to the respective community background and community action projects in the results and discussion. All authors contributed to the article and approved the submitted version.
